# Anterior chest wall in SAPHO syndrome: magnetic resonance imaging findings

**DOI:** 10.1186/s13075-020-02309-6

**Published:** 2020-09-14

**Authors:** Meiyan Yu, Yihan Cao, Junqiu Li, Yanan Zhang, Yuqian Ye, Lun Wang, Ziwei Huang, Xinyu Lu, Chen Li, Jianwei Huo

**Affiliations:** 1 Department of Radiology, Beijing Hospital of Traditional Chinese Medicine, Capital Medical University, Mei Shu Guan Hou Street, Beijing, 100010 China; 2 Department of Radiology, Peking Union Medical College Hospital, Peking Union Medical College and Chinese Academy of Medical Sciences, Beijing, 100730 China; 3 Institute of Clinical Medicine, Peking Union Medical College and Chinese Academy of Medical Sciences, Beijing, 100730 China; 4 School of Traditional Chinese Medicine, Beijing University of Traditional Chinese Medicine, Beijing, 100029 China; 5 Department of Traditional Chinese Medicine, Peking Union Medical College Hospital, Peking Union Medical College and Chinese Academy of Medical Sciences, No.1, Shuai Fu Yuan, Beijing, 100730 China

**Keywords:** SAPHO syndrome, Magnetic resonance imaging, Anterior chest wall, Bone marrow edema, Enthesitis, Synovitis

## Abstract

**Background:**

The anterior chest wall (ACW) involvement is characteristic of synovitis, acne, pustulosis, hyperostosis, and osteitis (SAPHO) syndrome, yet little research has focused on its magnetic resonance imaging (MRI) findings.

**Purpose:**

To characterize the MRI features of the ACW in patients with SAPHO syndrome.

**Methods:**

Seventy-one patients with SAPHO syndrome and ACW involvement evidenced by bone scintigraphy were recruited in this cross-sectional study. The ACW region was scanned using sagittal, axial, and oblique coronal Dixon T2-weighted sequences and axial Dixon T1-weighted sequences. The characteristics of both active inflammatory and chronic structural lesions were evaluated.

**Results:**

The ACW lesions exhibited an asymmetrical distribution and a predilection for the sternocostoclavicular region (93.0%). Notably, 91.5% of the patients had lesions in the area of the anterior first ribs. Bone marrow edema (BME) was observed in 63 (88.7%) patients, which mainly affected the sternocostal joints (87.3%) and the manubrium sterni (84.5%). All of the BMEs were distributed under the articular surface or the bone cortex, consistent with the distribution of the ligaments and joint capsules. Synovitis was detected in 64 (90.1%) patients, with a predilection for the sternoclavicular joints (76.1%). A soft tissue mass or infiltration was found in all the patients who had bone marrow edema. Thirteen (18.3%) patients showed venous stenosis. Structural changes included bone bridge formation (80.3%), hyperostosis (43.7%), and fat infiltration (39.4%). Four common patterns of involvement were observed: the first rib area, the sternoclavicular area, the sternal angle area, and the areas of the second to sixth sternocostal joints.

**Conclusion:**

The ACW lesions of SAPHO syndrome demonstrated a triad of enthesitis, synovitis, and osteitis, suggesting complex interactions among the ligaments, synovium, and bones in the region. The inflammatory changes in the first rib area were highlighted in SAPHO syndrome.

## Summary statement

The anterior chest wall involvement of SAPHO syndrome demonstrated a triad of enthesitis, synovitis, and osteitis, with prominent lesions in the first rib area.

## Key results

- The lesions of the anterior chest wall exhibited an asymmetrical distribution and a predilection for the area of the anterior first ribs.
- All of the bone marrow edemas were distributed under the articular surface or the bone cortex, consistent with the distribution of the ligaments and joint capsules.
- Four common patterns of involvement were observed: the first rib area, the sternoclavicular area, the sternal angle area, and the areas of the second to sixth sternocostal joints.

SAPHO syndrome: synovitis, acne, pustulosis, hyperostosis, and osteitis syndrome

## Introduction

Synovitis, acne, pustulosis, hyperostosis, and osteitis (SAPHO) syndrome, first proposed by Chamot et al. in 1987, is a spectrum of heterogeneous diseases characterized by osteoarticular and dermatological manifestations [1]. This disease is distributed globally, with an estimated annual prevalence of less than 1 in 10,000 for Caucasians and 1.44 in 1 × 10^8^ in individuals of Japanese descent [2]. Diagnostic challenges may arise due to heterogeneous manifestations of the disease: either acne and arthritis or acne and anterior chest wall (ACW) osteitis with an unclear pustulosis history [3]. The ACW is the most commonly involved site, affecting approximately 70–90% of adult patients [4–9]. The radiological findings of the osteoarticular manifestations in SAPHO syndrome play a key role in its early and correct diagnosis [10]. The typical “bull’s head” change on whole-body bone scintigraphy (WBBS) is characteristic of SAPHO syndrome [4], but the frequency of the sign is low [5]. Conventional X-ray and computed tomography examinations have limited capacity in visualizing active inflammatory changes [4]. Magnetic resonance imaging (MRI) is the only method that can detect bone marrow edema (BME) and soft tissue involvement [11]. However, only a few prior reports have specifically investigated the MRI abnormalities of the ACW in SAPHO patients [12–15]. The aim of our study was to characterize the MRI findings of ACW involvement in SAPHO patients so as to facilitate the understanding of the imaging features and pathogenesis of the disease.

## Methods

### Patients and clinical evaluation

In this cross-sectional study, seventy-one patients fulfilling the diagnostic criteria for SAPHO syndrome proposed by Kahn et al. [9] were enrolled in Peking Union Medical College Hospital and Beijing Traditional Chinese Medicine Hospital from March 2018 and June 2019. All patients were aged 18–70 and had ACW involvement confirmed by WBBS using ^99m^Tc-MDP. ACW involvement was defined by abnormally increased tracer uptake in the ACW region delineated superiorly by the suprasternal notch, sternoclavicular joints (SClJs), and clavicles, inferiorly by the costal arch, and laterally by the anterior axillary line. Informed written consent was obtained from each patient.

Demographic data and clinical features were recorded, including osteoarticular symptoms and dermatological manifestations. Laboratory evaluations included erythrocyte sedimentation rate (ESR), high-sensitivity C-reactive protein (hs-CRP), rheumatoid factor (RF), anti-nuclear antibody (ANA), and human leukocyte antigen (HLA)-B27, which were measured within 3 days of MRI examination. The Ethics Committee of Beijing Hospital of Traditional Chinese Medicine, Capital Medical University, approved this study (ethics document number: 2018BL-0-050-01).

### MRI protocol

MR imaging of the ACW region was performed on a Magnetom Skyra 3.0 T scanner (Siemens Healthcare, Germany). Patients were imaged in the supine position using a surface phased-array coil. Respiratory-triggered sagittal, axial, and oblique coronal T2W Turbo Spin Echo (TSE) Dixon MRI sequences were initially performed, followed by an axial T1W Dixon-Volume Interpolation Breathhold Examination (VIBE) sequence. VIBE sequence was adopted to reduce scan time as prolonged scan duration is particularly uncomfortable for SAPHO patients. Briefly, the following parameters were used for the T2W TSE Dixon sequences: repetition time (TR) 2500 ms and echo time (TE) 92 ms for sagittal images, TR 3500 ms and TE 92 ms for oblique coronal and axial images, field of view (FOV) 260 mm, imaging matrix 205 mm × 256 mm, section thickness 3 mm, and interslice gap 0.3 mm. For the axial T1W Dixon-VIBE sequence, the parameters were TR 4.11 ms, TE 1.31 ms, FOV 380 mm, imaging matrix 195 × 320 mm, section thickness 3 mm, and interslice gap 0.3 mm.

### Analysis of images

All images were evaluated independently by two radiologists (ZYN: 6 years of MRI experience; LJQ: 25 years of MRI experience) blinded to the clinical features and bone scintigraphy at the time of their reviews. Disagreements were resolved in a consensus reading with a third experienced musculoskeletal radiologist (HJW: 28 years of experience). The reviewers evaluated and recorded each anatomical site in the AWC for the presence of the following features: (1) BME, defined as a hyperintense bone marrow signal on water-only T2W Dixon images on two consecutive slices. The symmetry and distribution of the lesions and the involvement of the soft tissue around the BME sites were also evaluated. (2) Synovitis, defined as the existence of high fluid accumulation signals in the joint cavity. (3) Fat infiltration, defined as bright bone marrow signals on fat-only T2W Dixon images on two consecutive slices. (4) Ossification of the costal cartilage, defined as eggshell-like continuous ossification or a wide-range ossification (see Fig. 4, as an example). (5) Hyperostosis, determined in contrast to the contralateral and surrounding bone. (6) Bone bridge, defined as an extended bone marrow signal connecting different bones [16–18]. (7) Venous stenosis, defined as a reduction in diameter compared with that of normal vessels [19].

In order to calculate the number of involved sites, we defined 18 sites in the ACW region: the SClJs, the costoclavicular ligament area (including the medial end of the clavicles), the middle and lateral region of the clavicles, the 1st sternocostal joints (SCoJs), the 2nd SCoJs and sternal angle, the 3rd SCoJs, the 4th SCoJs, the 5th SCoJs, and the 6th and 7th SCoJs. Each area was further divided into the left and right sites.

### Statistics

Data were presented as numbers (%) for categorical variables and mean (SD) for continuous variables. Agreement was expressed in Cohen’s *κ*, positive agreement, and negative agreement. Student’s *t* test was applied to compare between groups for continuous variables. All tests were two-tailed with the significant level of 0.05. Analyses were conducted using R 3.5.3 and SPSS 19.0.

## Results

### Demographic and clinical characteristics

There were 71 patients (55 women and 16 men, aged 23 to 69 years old) included in our study. The mean age of onset of osteoarticular symptoms and skin lesions were 49.0 ± 10.6 and 40.2 ± 10.2 years. The mean age of diagnosis was 44.0 ± 12.3 years and the mean disease duration was 51.3 ± 9.9 months. All patients had suffered anterior chest pain in the course of the disease. Palmoplantar pustulosis was the most common skin lesion (83.1%), and severe acne appeared in 7.0% of patients. Only 9.9% of patients had no skin lesions. None of the patients had swelling of arm or neck or other symptoms of central venous stenosis. There were 63.4% of patients with elevated ESR or hs-CRP. The RF, ANA, and HLA-B27 were positive in 7.0%, 2.8%, and 5.6% of patients, respectively. The details are provided in Table 1.

**Table 1:** Demographic and clinical characteristics of the 71 patients with SAPHO syndrome

Demographic and clinical characteristics (***n*** = 71)	
**Demographic characteristics**	
Sex, female/male	55/16
Age at imaging, mean (S.D.), years	44.0 (12.1)
Age at onset of osteoarticular symptoms, mean (S.D.), years	49.0 (10.6)
Age at onset of skin lesions, mean (S.D.), years	40.2 (10.2)
Duration of disease, mean (S.D.), months	51.3 (9.9)
Age at SAPHO diagnosis, mean, (S.D.), years	44.0 (12.3)
Duration of diagnosis, mean (S.D.), years	4.0 (5.0)
**Clinical characteristics**, number (%)	
Anterior chest pain	71 (100.0)
Spinal or sacroiliac pain	52 (73.2)
Peripheral skeletal pain	41 (57.7)
Skin manifestations	
None	7 (9.9)
Palmoplantar pustulosis	59 (83.1)
Severe acne	5 (7.0)
**Laboratory findings**	
ESR, median (range), mm/h	17 (2–91)
hs-CRP, median (range), g/l	4.24 (0.41–57.63)
ESR or hs-CRP elevated, number (%)	45 (63.4)
ANA positive, number (%)	5 (7.0)
RF positive, number (%)	2 (2.8)
HLA-B27 positive, number (%)	4 (5.6)
**Whole-body bone scintigraphy**	
Anterior chest wall	71 (100)
Spine	43 (60.6)
Sacroiliac joint	28 (39.4)
Peripheral skeleton	22 (31.0)
Skull	9 (12.7)
Pelvis	3 (4.2)

*ESR* erythrocyte sedimentation rate, *hs-CRP* high-sensitivity C-reactive protein, *ANA* antinuclear antibody, *RF* rheumatoid factor, *HLA-B27* human leukocyte antigen-B27

### MRI findings of ACW

The agreement between the two readers (ZYN and LJQ) was sufficient. Among the 55 independent variables of MRI features, 48 (87.3%) variables had an interrater agreement ≥ 0.7 between ZYN and LJQ and 7 (12.7%) had an interrater agreement 0.6 to 0.7 (see Supplementary 1).

All 71 patients exhibited abnormalities on ACW MRI. On average, 4.8 ± 3.4 sites were involved per patient with a median of 4 and a range of 1 to 17. Male patients tended to have more involved sites than female patients though the difference was not statistically significant (6.1 ± 4.0 vs. 4.4 ± 3.1, *P* = 0.061). The lesions exhibited a mixture of active (inflammatory) and chronic (structural) changes, with an asymmetrical distribution and a predilection for the sternocostoclavicular region (93.0%), including the SClJs (76.1%) and the sternocostal joints (69%). For patients with lesions in all the three areas (sternal, costal, and clavicular regions), unilateral involvement was predominant, with 24 (33.8%) and 19 (26.8%) cases on the left and right side, respectively. Bilateral lesions were only seen in 13 (18.3%) patients. Notably, 91.5% patients had lesions in the anterior first rib and its surrounding soft tissue.

#### BME

**Fig. 1:**
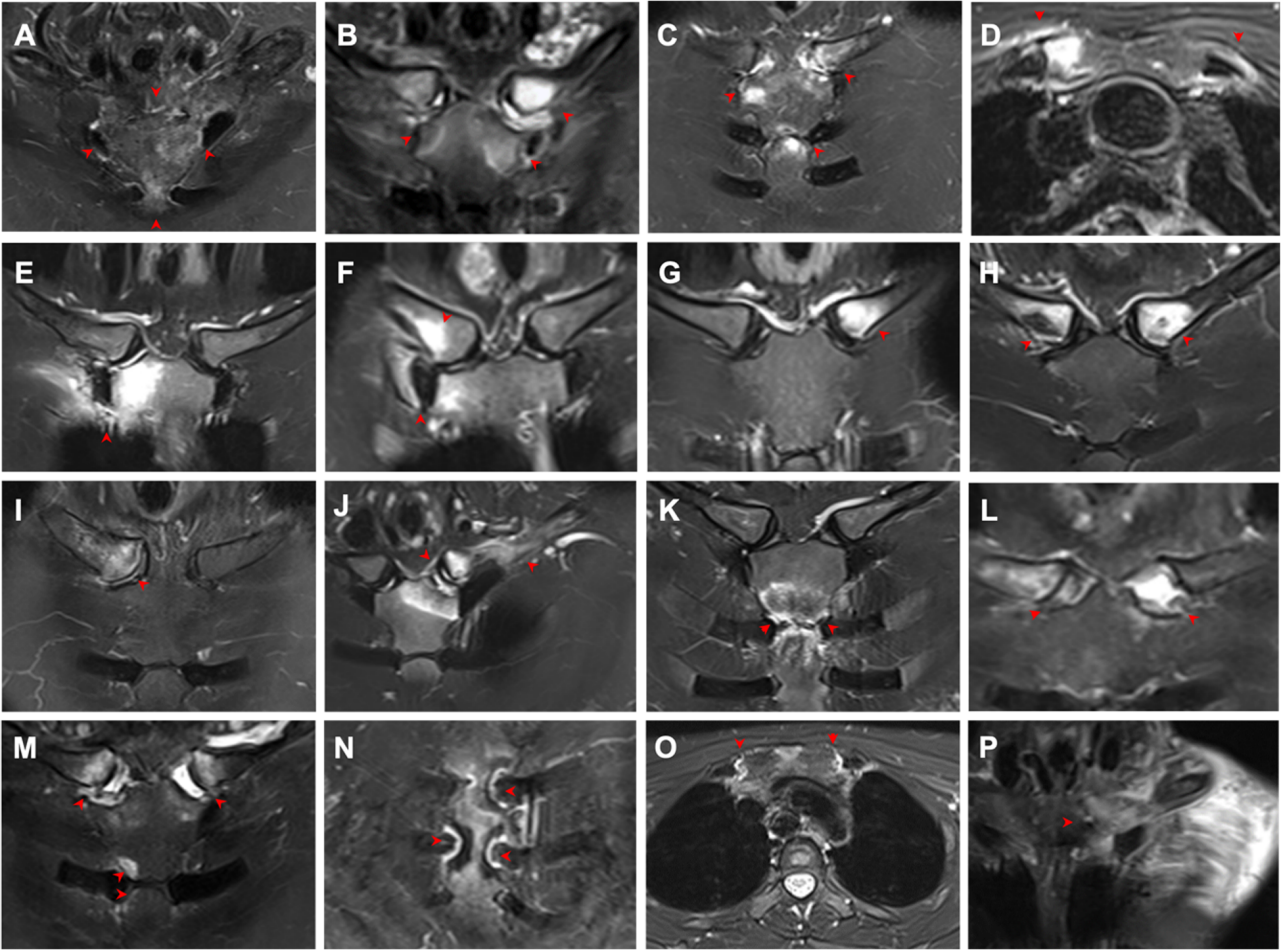
Active inflammatory lesions in the ACW shown by oblique coronal (**a**–**c**, **e**–**n**, **p**) and axial (d, o) water-only T2W Dixon images in patients with SAPHO syndrome. **a** Extended BME in the bilateral sternoclavicular region, resembling the “bull’s head” sign. **b** BME of the sternocostoclavicular region. **c** BME of the sternocostoclavicular region and the sternal angle. **d** BME of the ossified bones in the first left rib and the first right SCoJ. **e** Active inflammatory changes of the surrounding tissue of the first right rib and the first right SCoJ. **f** Active inflammatory changes of the right costoclavicular ligament area and the first right SCoJ. **g** BME of the medial end of the left clavicle. **h** BME of the medial ends of the bilateral clavicles. **i** BME and hyperostosis of the medial end of the right clavicle. **j** Active inflammatory changes of the left costoclavicular ligament area and the SClJ. **k** BME of the sternal angle. **l** Pseudowidening of the SClJ. **m** BME of the bilateral SClJs with pseudowidening of joint space and BME of the right side of the sternal angle. **n** Joint effusion in the bilateral second and third SCoJ. **o** Joint effusion in the bilateral first SCoJ and soft tissue mass behind the manubrium sterni. **p** Soft tissue infiltration in the chest wall. Lesions are indicated by red arrows. BME, bone marrow edema; SCoJ, sternocostal joint; SClJ, sternoclavicular joint

**Fig. 2:**
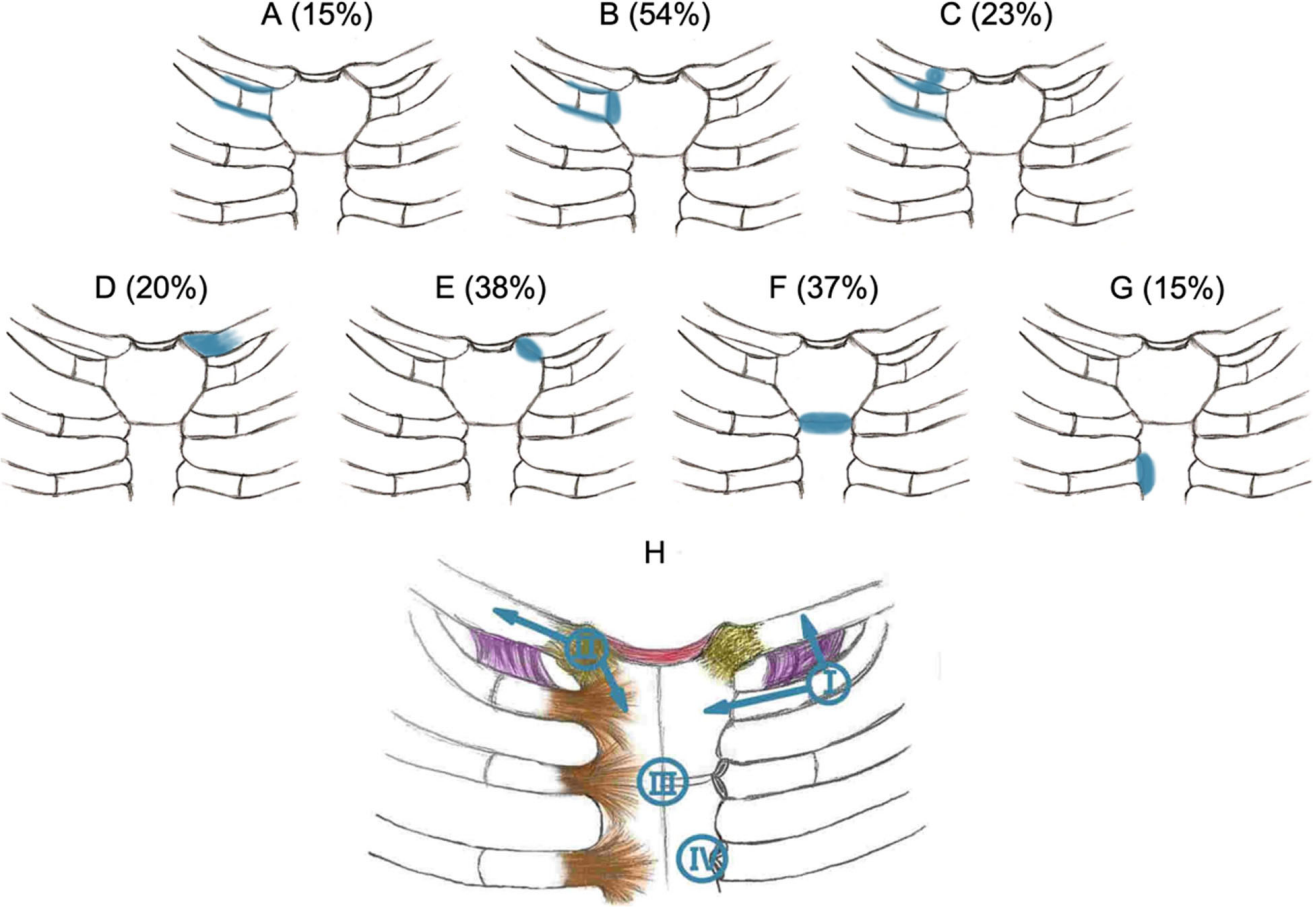
Distribution of bone marrow edema and edema of the surrounding soft tissue in the ACW region in patients with SAPHO syndrome. **a** The first rib lesion without involvement of the first SCoJ or the medial end of the clavicle. **b** The first SCoJ lesion. **c** Lesions in the costoclavicular ligament area with involvement of the medial end of the clavicle. **d** Lesions in the medial end of the clavicle adjacent to the manubrium without involvement of the adjacent sternoclavicular joint. **e** The SClJ lesion. **f** The sternal angle lesion. **g** Lesions in the second to sixth SCoJ. **h** The four common patterns of involvement: (I) the first rib area, possibly developing from the anterior end of the first rib (type A) to the first SCoJ (type B) and the costoclavicular ligament (type C); (II) the sternoclavicular area, involving the SClJ (type E), the medial end of the clavicle (type D), and the interclavicular ligament; (III) the sternal angle area (type F); and (IV) the areas of the second to sixth SCoJs (type G). BME, bone marrow edema; SCoJ, sternocostal joint; SClJ, sternoclavicular joint

BME, which indicated acute inflammation, was observed in 63 (88.7%) patients. Affected sites included the manubrium sterni (60, 84.5%), sternal angle (26, 36.6%), mesosternum (11, 15.5%), clavicle (35, 49.3%), and second rib (2, 2.8%). All the involved sites showed signs of inflammation in the surrounding soft tissues. As for the joints, the most commonly affected sites were the SCoJs (87.3%), followed by the SClJs (38.0%). Extended BME involving the sternum and bilateral SClJs (resembling the “bull’s head” sign on bone scintigraphy) was revealed in only 3 (4.2%) patients. All of the BMEs were distributed under the articular surface or the cortex, consistent with the distribution of ligaments and joint capsules (Table 2 and Fig. 1 a, b, e). Four common patterns of involvement were observed (Fig. 2): (I) the first rib area, possibly developing from the anterior end of the first rib to the first SCoJ and the costoclavicular ligament; (II) the sternoclavicular area, involving the SClJ, the medial end of the clavicle, and the interclavicular ligament; (III) the sternal angle area; and (IV) the areas of the second to sixth SCoJs.

**Table 2:** Active lesions in the anterior chest wall detected by MRI in the 71 patients with SAPHO syndrome

Active lesions	Number (%)
**Bone marrow edema**	
Sternoclavicular joint	27 (38.0)
Left only	9 (12.7)
Right only	6 (8.5)
Bilateral	12 (16.9)
Sternocostal joint	62 (87.3)
Left only	7 (9.9)
Right only	12 (16.9)
Bilateral	43 (60.6)
1st	38 (53.5)
2nd to 6th	11 (15.5)
Clavicle	35 (49.3)
Medial end, left only/right only/bilateral	11 (15.5)/8 (11.3)/16 (22.5)
Middle and lateral region, left only/right only/bilateral	1 (1.4)/1 (1.4)/0 (0)
Sternum	
Manubrium sterni	60 (84.5)
Region next to the clavicle and the first rib	54 (76.1)
Left only/right only/bilateral	10 (14.1)/9 (12.7)/35 (49.3)
Sternal angle	26 (36.6)
Mesosternum	11 (15.5)
Region next to the 3rd ribs	8 (11.3)
Region next to the 4th ribs	7 (9.9)
Region next to the 5th ribs	6 (8.5)
Region next to the 6–8th ribs	7 (9.9)
Xiphoid	3 (4.2)
**Synovitis**	
Sternoclavicular joint	54 (76.1)
Left only/right only	4 (5.6)/8 (11.3)
Bilateral	43 (60.6)
Narrowing of joint space, left only/right only/bilateral	1 (1.4)/2 (2.8)/8 (11.3)
Pseudowidening of joint space, left only/right only/bilateral	2 (2.8)/1 (1.4)/7 (9.9)
Manubriosternal joint	5 (7.0)
Sternocostal joint	11 (15.5)
1st, left only/right only/bilateral	0 (0)/0 (0)/2 (2.8)
2nd, left only/right only/bilateral	2 (2.8)/2 (2.8)/7 (9.9)
3rd, left only/right only/bilateral	1 (1.4)/4 (5.6)/5 (7.0)
4–6th, left only/right only/bilateral	0 (0)/0 (0)/4 (5.6)

#### Synovitis

Joint effusion on MR images was recognized in 64 (90.1%) patients, with a predilection for the SClJs (76.1%), followed by the SCoJs (15.5%), including the first SCoJ (2.8%). Joint effusion was also found in the manubriosternal joint (MSJ) (7.0%), which was reported to be a synovial joint in ~ 30% of patients [20] (Table 2 and Fig. 1 f, g, h).

#### Soft tissue involvement

**Fig. 3:**
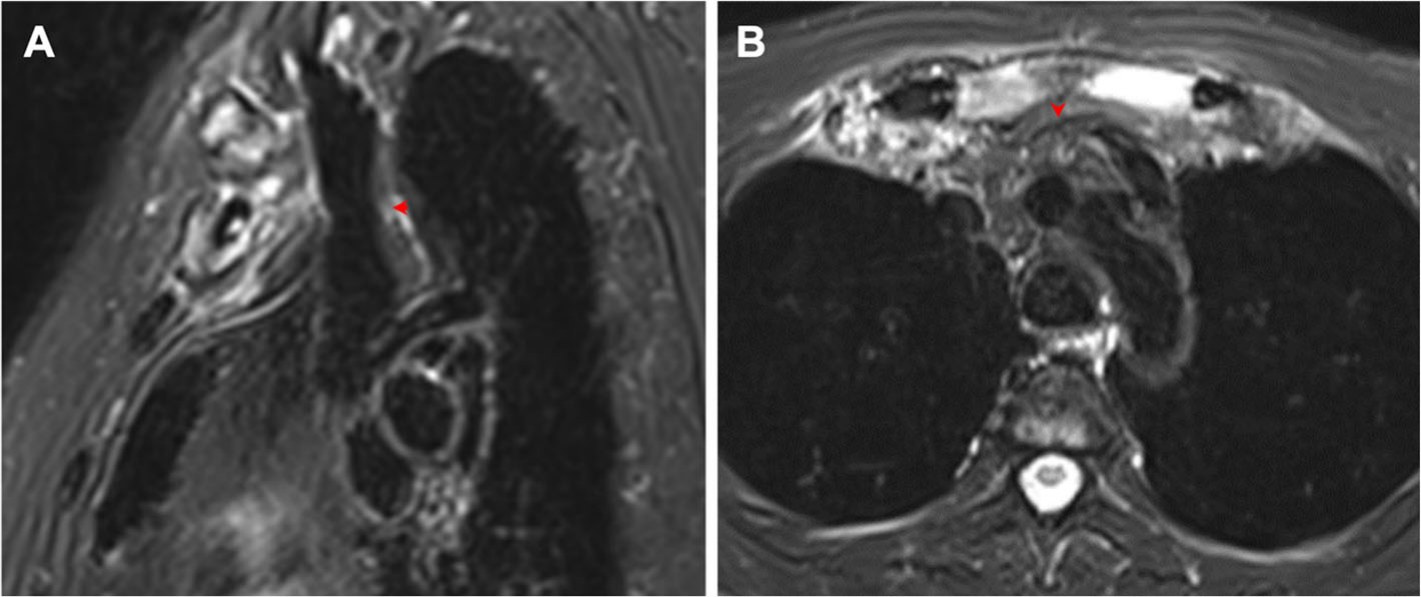
Soft tissue mass in the ACW region. **a** Sagittal water-only T2W Dixon image shows a soft tissue mass or infiltration around the hyperostotic bones in a 55-year-old female with a disease duration of 60 months. The lower part of the superior vena cava was normal, but the right brachiocephalic vein behind the soft tissue mass was slightly narrowed (red arrow). **b** Axial water-only T2W Dixon image reveals a soft tissue mass or infiltration behind the manubrium sterni in a 49-year-old male with a disease duration of 6 months. The left brachiocephalic vein was surrounded by the soft tissue mass and could not be evaluated (red arrow)

A soft tissue mass or infiltration was observed in all the patients who had BME, among which 49 (69.0%) patients exhibited a retrosternal tumor-like appearance. We found 16 (22.5%) patients with edema in the pectoralis major, most in diffused directions or in the direction of the myofibers. Venous stenosis was identified in 13 (18.3%) patients, including the right (6, 8.8%) and left (12, 16.9%) brachiocephalic veins (Fig. 1 h, i and Fig. 3).

#### Findings of the first ribs

**Fig. 4:**
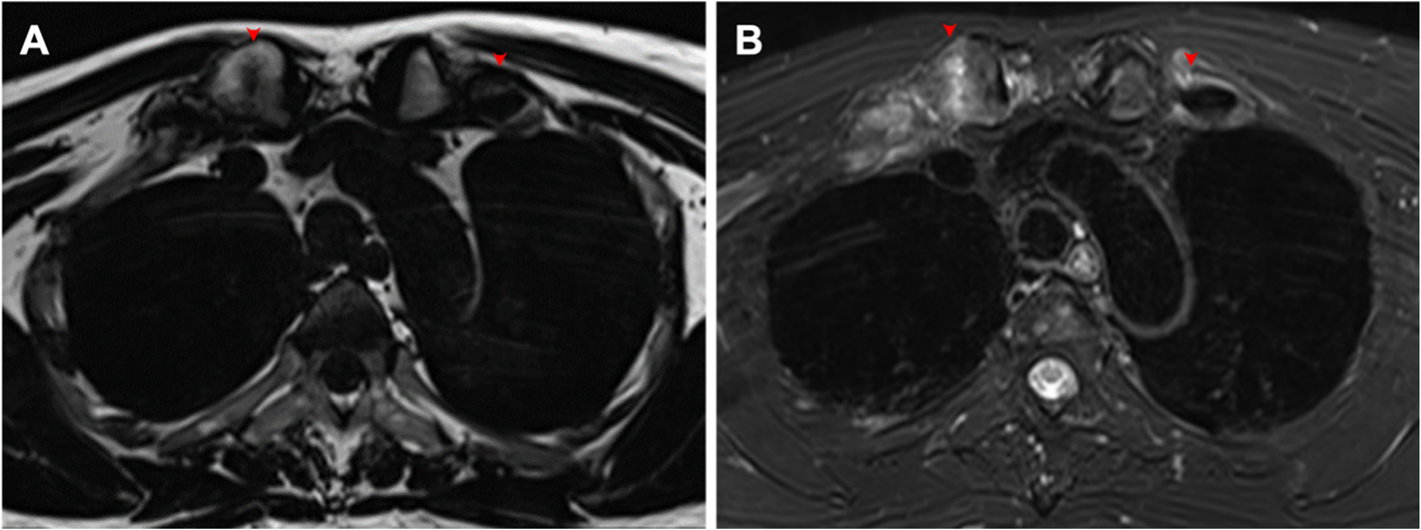
Lesions of the first costal cartilage in a 43-year-old female with SAPHO syndrome. **a** Axial fat-only T2W Dixon image shows irregular hyperintensity in the ossified bones in the right first costal cartilage, indicating fat infiltration. **b** Axial water-only T2W Dixon image shows irregular ossification beneath the perichondrium of the bilateral first costal cartilage and hyperintensity in the surrounding soft tissue, indicating inflammation. Lesions are indicated by red arrows

In our patients, the manifestation of the first ribs was interesting and is therefore elaborated here specifically. Sixty-five (91.5%) patients had lesions in the anterior first ribs and the surrounding soft tissue. Thirty-seven (52.1%) patients had different degrees of ossification in the first rib, with 8 (11.3%) and 11 (15.5%) cases on the left and right side, respectively. Bilateral ossification was observed in 18 (25.4%) patients. Notably, 70% of ossified first ribs had BME, 22% had fat infiltration, and 50% had asymmetrical hyperostosis (Fig. 4).

#### Chronic structural lesions

**Fig. 5:**
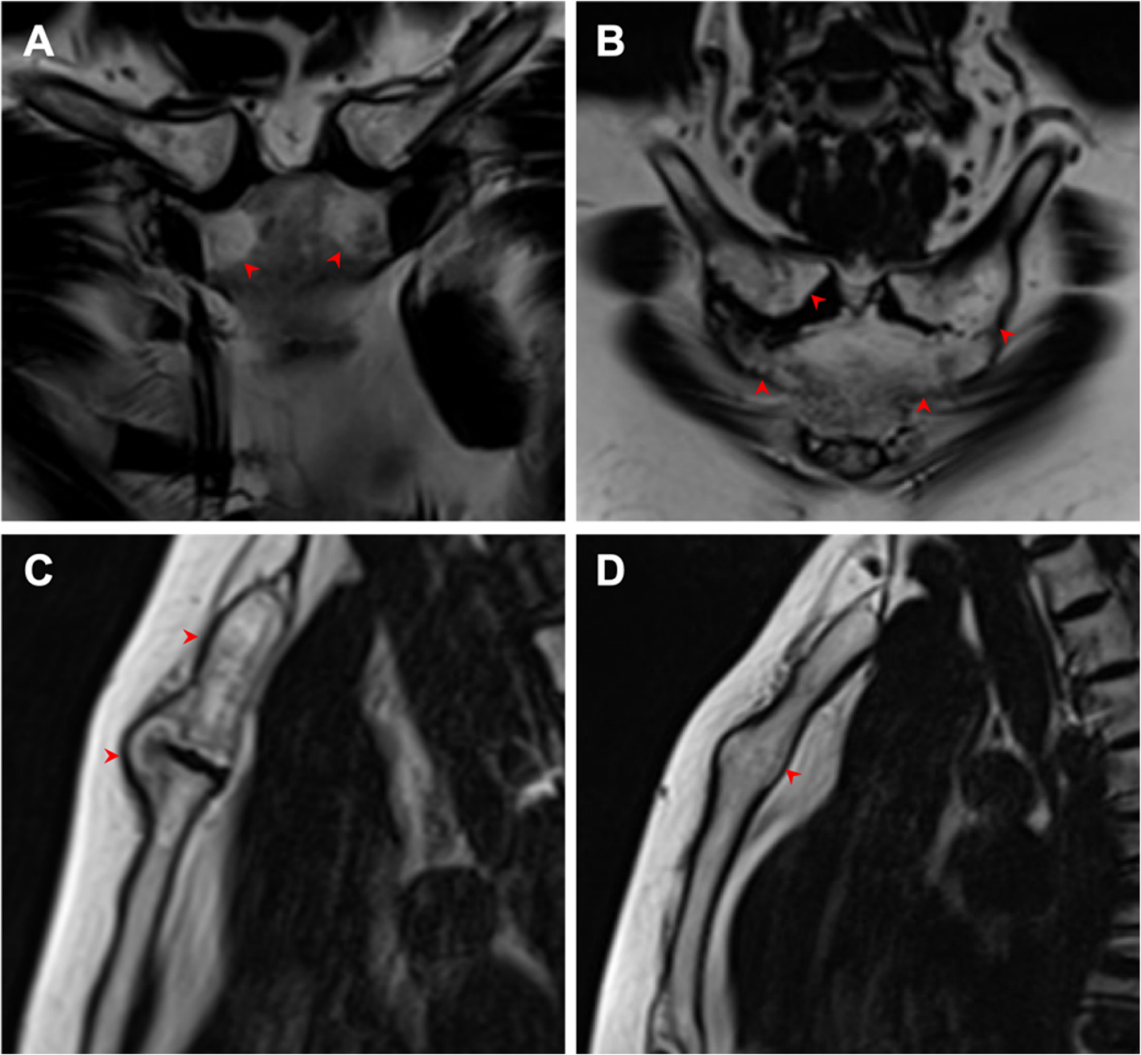
Chronic lesions in the ACW shown by oblique coronal (**a**–**c**) and sagittal (**d**, **e**) T2W Dixon images in patients with SAPHO syndrome. **a** Fat-only T2W Dixon image shows different shapes of fat infiltration, such as crescent in the corners of the manubrium, sheet in the right clavicle, and scattered lesions in the left clavicle. **b** Fat-only T2W Dixon image shows ossification of the costoclavicular ligament. **c** Fat-only T2W Dixon image shows a bone bridge of the sternal angle. **d** Fat-only T2W Dixon image shows hyperostosis in the sternal angle. Lesions are indicated by red arrows

Different types of chronic lesions were detected. Bone bridges were observed in 80.3% patients, hyperostosis in 43.7% patients, and fat infiltration in 39.4% patients. Hyperostosis was widespread, observed in the clavicle (26.8%), manubrium sterni (31.0%), sternal angle (31.0%), and mesosternum (8.5%). Fat infiltration was distributed widely in the manubrium sterni (38.0%) and mesosternum (12.7%). There were different shapes of fat infiltration, such as crescent (9.9%), sheet (14.1%) and scatter (31%) (Table 3 and Fig. 5).

**Table 3:** Chronic lesions in the anterior chest wall detected by MRI in the 71 patients with SAPHO syndrome

Chronic lesions	Number (%)
**Bone bridge**	57 (80.3)
Costoclavicular ligament	11 (15.5)
Left only	0 (0)
Right only	4 (5.6)
Bilateral	7 (9.9)
Radiate sternocostal ligament (the 1st rib)	55 (77.5)
Left only	1 (1.4)
Right only	1 (1.4)
Bilateral only	53 (74.6)
Sternoclavicular ligament	11 (15.5)
Left only	0 (0)
Right only	4 (5.6)
Bilateral	7 (9.9)
Sternal angle	17 (23.9)
**Hyperostosis**	31 (43.7)
Clavicle	19 (26.8)
Left only	2 (2.8)
Right only	7 (9.9)
Bilateral	10 (14.1)
Manubrium sterni	22 (31.0)
Sternal angle	22 (31.0)
Mesosternum	6 (8.5)
**Fat infiltration**	28 (39.4)
Manubrium sterni	27 (38.0)
Mesosternum	9 (12.7)

## Discussion

The ACW is the most predominantly involved area of SAPHO syndrome in adults. MRI enables the detection of both active (inflammatory) and chronic (structural) changes in this region with a high sensitivity and level of detail [4, 20, 21].

Any areas of the ACW can be affected, but the sternoclavicular, manubriosternal, and sternocostal joints are the most commonly involved sites according to previous studies [4, 9, 11, 22]. In our study, the most frequently involved sites were the SClJ and the first rib area. Such findings were consistent with a study by Cao et al. based on ^99m^Tc-MDP WBBS [5].

The pathogenesis and evolvement of ACW lesions in SAPHO syndrome remains unclear. The osteoarticular changes in the ACW were subdivided into three stages based on conventional radiography [23]. Stage 1 is mild ossification localized to the costoclavicular ligament. Stage 2 is generalized ossification of the sternocostoclavicular region. Stage 3 is a further progression of the hyperostotic changes, involving the superior margin of the clavicle. This development suggests a disease primarily involving entheses, particularly at the costoclavicular ligament, that spreads to the adjacent joints and bones. However, changes may also begin in the bones, often in the manubrium sterni, and spread to the joints and surrounding capsular and ligamentous structures [6]. Two theories (inflammatory enthesitis and reactive osteitis elicited by slow microorganism infection) regarding the pathology of the disease have also been proposed [24].

Our study identified several characteristics of ACW lesions on MRI that might unveil the pathogenic process of SAPHO syndrome. First, a triad of enthesitis, synovitis, and osteitis was demonstrated. Ligamentous involvement was directly evidenced by bone bridge formations along the ligaments. Second, all of the BMEs were distributed under the articular surface or the cortex, consistent with the distribution of ligaments and joint capsules. The BMEs seem to involve any area attached to ligaments and synovial joints, not necessarily initiating from the costoclavicular ligament as proposed by Sonozaki et al. [20]. Given that all the BMEs were related to the insertion sites of the ligaments and joint capsules, we speculate that enthesitis might be the primary disorder. Similar findings were observed in studies on spinal MRI and CT, which indicate that vertebral involvement in SAPHO syndrome may be triggered by vertebral corner lesions originating from the enthesitis at the junction of the outer fibers of the annulus fibrosis and vertebral epiphyseal ring [24, 25].

Why were the first rib area and the SClJs the most commonly affected sites in the ACW? Anatomically, the first costal cartilage can ossify in adolescence, so the internal environment is relatively complex [26, 27]. In addition, the costoclavicular ligaments and the radiate sternocostal ligaments are related to the first ribs [28]. The SClJ, a true diarthrodial synovial joint, has attachments to three complex ligaments (the interclavicular ligament, anterior sternoclavicular ligament, and posterior sternoclavicular ligament) [28]. However, synovitis of the ACW has been rarely reported [29]. In our study, 90.1% of patients showed effusion in the joint cavities, although some of the manifestation may be physiological effusions, which should be proved by enhanced MRI [30, 31]. In addition, the sternal angle, which is in the vicinity of the second SCoJ (containing an interarticular ligament and two synovial membranes) and contains the manubriosternal joint (containing dorsal and ventral ligaments and classified as a synovial joint in ~ 30% patients [20]), was the third-most frequently involved site.

In summary, the complex anatomical structure in the ACW region may contribute to the patterns of lesions in SAPHO syndrome on MRI. BME can affect any site related to ligaments and synovial joints in solitary or mixed patterns (Fig. 2 a–g). We observed four common patterns of involvement (Fig. 2 h): the first rib area, the sternoclavicular area, the sternal angle area, and the areas of the second to sixth SCoJ. These patterns can occur alone or in combinations. Further longitudinal follow-up studies are needed to investigate the evolvement of these lesions.

Structural changes may occur during periods of remission, when bone erosion tends to heal with sclerosis and periosteal new bone formation [6, 11]. Such changes are best revealed by CT imaging. In our study, we preliminarily evaluated hyperostosis and bone bridges using MRI. Further validation of the results on CT is needed. We also found that fat infiltration was widely distributed with various shapes in the ACW region, which was considered to develop from BME during remission periods [7].

Inflammatory involvement of adjacent soft tissue was often present. Studies have emphasized that voluminous soft tissue may compress or obstruct the subclavian vein, causing thoracic outlet syndrome [8, 32, 33]. Our analysis found that 13 patients had stenosis of the brachiocephalic veins, all of whom were asymptomatic. As non-enhanced images were not suitable for vascular evaluation, the severity of the stenosis was not assessed. Furthermore, the pectoralis major showed obvious edema, which may mimic an aggressive process such as lymphoma or other malignancies. However, there was no delineated soft tissue mass or signs suggesting abscess formation.

There were some limitations to our study. First, there was no control group. Some other diseases may involve the ACW, such as osteoarthritis, septic arthritis, and other inflammatory conditions [20, 34]. Inclusion of control groups may help with better interpretation of the findings in SAPHO syndrome. Second, the non-contrast-enhanced MRI could not show all the involved veins, such as subclavian veins. The sensitivity and specificity for identifying synovitis and tenosynovitis are also lower using T2-weighted sequences alone compared with contrast-enhanced images [30]. Third, there was no computed tomography serving as a reference to accurately assess ossification of the costal cartilage and ligaments. Fourth, the patients were heterogeneous in terms of disease duration and treatment. Most patients had a complex medical history and treatment process, which may lead to heterogeneous manifestations [15]. Last, future studies should incorporate patient reported outcomes (such as magnitude of pain and upper extremity function) to better understand the clinical relevance of the MRI findings.

## Conclusions

The ACW lesions of SAPHO syndrome demonstrated a triad of enthesitis, synovitis, and osteitis, suggesting complex interactions among the ligaments, synovium, and bones in the region. The inflammatory changes in the first rib area were highlighted in SAPHO syndrome.

## Supplementary information

**Additional file 1: Supplementary 1.** Interrater agreement of the MRI features.
