# Supplementary material for: Anterior chest wall in SAPHO syndrome: magnetic resonance imaging findings
Source: Arthritis Res Ther. 2020 Sep 14;22:216. doi: 10.1186/s13075-020-02309-6 (PMC7491189; doi:10.1186/s13075-020-02309-6)
Supplement: Supplementary file 1 — Additional file 1: Supplementary 1. Interrater agreement of the MRI features. [file 13075_2020_2309_MOESM1_ESM.docx]

**Supplementary 1. Interrater agreement of the MRI features**

| **Variables** | **Rater 1^*^** | **Rater 2^*^** | **κ** | **positive agreement** | **negative agreement** |
| --- | --- | --- | --- | --- | --- |
| Fat infiltration, manubrium | 27 (38.0) | 30 (42.3) | 0.91 | 94.7 | 96.5 |
| Fat infiltration, mesosternum | 9 (12.7) | 11 (15.5) | 0.88 | 90.0 | 98.4 |
| Hyperostosis, left clavicle | 10 (14.1) | 13 (18.3) | 0.84 | 87.0 | 97.5 |
| Hyperostosis, right clavicle | 16 (22.5) | 18 (25.4) | 0.92 | 94.1 | 98.1 |
| Hyperostosis, manubrium | 18 (25.4) | 24 (33.8) | 0.80 | 85.7 | 94.0 |
| Hyperostosis, sternal angle | 20 (28.2) | 25 (35.2) | 0.84 | 88.9 | 94.8 |
| Hyperostosis, mesosternum | 4 (5.6) | 6 (8.5) | 0.79 | 80.0 | 98.5 |
| Bone bridge, left sternoclavicular ligament | 7 (9.9) | 8 (11.3) | 0.93 | 93.3 | 99.2 |
| Bone bridge, right sternoclavicular ligament | 9 (12.7) | 10 (14.1) | 0.82 | 84.2 | 97.6 |
| Bone bridge, left radiate sternocostal ligament (the 1st rib) | 53 (74.6) | 54 (76.1) | 0.73 | 93.5 | 80.0 |
| Bone bridge, right radiate sternocostal ligament (the 1st rib) | 53 (74.6) | 56 (78.9) | 0.80 | 95.4 | 84.8 |
| Bone bridge, left costoclavicular ligament | 8 (11.3) | 8 (11.3) | 0.86 | 87.5 | 98.4 |
| Bone bridge, right costoclavicular ligament | 11 (15.5) | 12 (16.9) | 0.84 | 87.0 | 97.5 |
| Bone bridge, sternal angle | 13 (18.3) | 20 (28.2) | 0.73 | 78.8 | 93.6 |
| Synovitis, left sternoclavicular joint, joint effusion | 46 (64.8) | 47 (66.2) | 0.91 | 96.8 | 93.9 |
| Synovitis, left sternoclavicular joint, narrowing of joint space | 8 (11.3) | 10 (14.1) | 0.87 | 88.9 | 98.4 |
| Synovitis, left sternoclavicular joint, pseudowidening of joint space | 8 (11.3) | 8 (11.3) | 0.86 | 87.5 | 98.4 |
| Synovitis, right sternoclavicular joint, joint effusion | 50 (70.4) | 53 (74.6) | 0.89 | 97.1 | 92.3 |
| Synovitis, right sternoclavicular joint, narrowing of joint space | 11 (15.5) | 12 (16.9) | 0.84 | 87.0 | 97.5 |
| Synovitis, right sternoclavicular joint, pseudowidening of joint space | 8 (11.3) | 7 (9.9) | 0.78 | 80.0 | 97.6 |
| Synovitis, left 1st sternocostal joint | 2 (2.8) | 1 (1.4) | 0.66 | 66.7 | 99.3 |
| Synovitis, right 1st sternocostal joint | 1 (1.4) | 2 (2.8) | 0.66 | 66.7 | 99.3 |
| Synovitis, sternal angle | 4 (5.6) | 5 (7.0) | 0.88 | 88.9 | 99.2 |
| Synovitis, left 2nd sternocostal joint | 8 (11.3) | 7 (9.9) | 0.78 | 80.0 | 97.6 |
| Synovitis, right 2nd sternocostal joint | 8 (11.3) | 9 (12.7) | 0.80 | 82.4 | 97.6 |
| Synovitis, left 3rd sternocostal joint | 6 (8.5) | 5 (7.0) | 0.70 | 72.7 | 97.7 |
| Synovitis, right 3rd sternocostal joint | 8 (11.3) | 9 (12.7) | 0.93 | 94.1 | 99.2 |
| Synovitis, left 4th sternocostal joint | 2 (2.8) | 3 (4.2) | 0.79 | 80.0 | 99.3 |
| Synovitis, right 4th sternocostal joint | 2 (2.8) | 3 (4.2) | 0.79 | 80.0 | 99.3 |
| Synovitis, left 5th sternocostal joint | 1 (1.4) | 2 (2.8) | 0.66 | 66.7 | 99.3 |
| Synovitis, right 5th sternocostal joint | 1 (1.4) | 2 (2.8) | 0.66 | 66.7 | 99.3 |
| Synovitis, left 6th sternocostal joint | 1 (1.4) | 2 (2.8) | 0.66 | 66.7 | 99.3 |
| Synovitis, right 6th sternocostal joint | 1 (1.4) | 2 (2.8) | 0.66 | 66.7 | 99.3 |
| BME, left sternoclavicular joint | 23 (32.4) | 22 (31.0) | 0.84 | 88.9 | 94.8 |
| BME, right sternoclavicular joint | 20 (28.2) | 22 (31.0) | 0.80 | 85.7 | 94.0 |
| BME, left sternocostal joint | 47 (66.2) | 51 (71.8) | 0.80 | 93.9 | 86.4 |
| BME, right sternocostal joint | 51 (71.8) | 56 (78.9) | 0.81 | 95.3 | 85.7 |
| BME, medial end of the left clavicle | 29 (40.8) | 26 (36.6) | 0.79 | 87.3 | 92.0 |
| BME, middle and lateral region of the left clavicle | 1 (1.4) | 1 (1.4) | 1.00 | 100 | 100 |
| BME, medial end of the right clavicle | 26 (36.6) | 24 (33.8) | 0.81 | 88.0 | 93.5 |
| BME, middle and lateral region of the right clavicle | 1 (1.4) | 1 (1.4) | 1.00 | 100 | 100 |
| BME, manubrium, region next to the left clavicle and 1st rib | 48 (67.6) | 46 (64.8) | 0.81 | 93.6 | 87.5 |
| BME, manubrium, region next to the right clavicle and 1st rib | 46 (64.8) | 46 (64.8) | 0.75 | 91.3 | 84.0 |
| BME, sternal angle | 29 (40.8) | 26 (36.6) | 0.91 | 94.5 | 96.6 |
| BME, mesosternum, region next to the 3rd ribs | 9 (12.7) | 8 (11.3) | 0.93 | 94.1 | 99.2 |
| BME, mesosternum, region next to the 4th ribs | 9 (12.7) | 7 (9.9) | 0.86 | 87.5 | 98.4 |
| BME, mesosternum, region next to the 5th ribs | 8 (11.3) | 7 (9.9) | 0.78 | 80.0 | 97.6 |
| BME, mesosternum, region next to the 6th ribs | 9 (12.7) | 6 (8.5) | 0.78 | 80.0 | 97.6 |
| BME, xiphoid | 3 (4.2) | 3 (4.2) | 1.00 | 100 | 100 |
| 1st rib involvement, soft tissue edema, left | 44 (62.0) | 47 (66.2) | 0.91 | 96.7 | 94.1 |
| 1st rib involvement, soft tissue edema, right | 49 (69.0) | 50 (70.4) | 0.83 | 94.9 | 88.4 |
| 1st rib involvement, ossification, left | 27 (38.0) | 24 (33.8) | 0.85 | 90.2 | 94.5 |
| 1st rib involvement, ossification, right | 30 (42.3) | 29 (40.8) | 0.91 | 94.9 | 96.4 |
| Venous stenosis, left brachiocephalic vein | 6 (8.5) | 12 (16.9) | 0.62 | 66.7 | 95.2 |
| Venous stenosis, right brachiocephalic vein | 5 (7.0) | 7 (9.9) | 0.82 | 83.3 | 98.5 |

^*^Data are presented as number (%). Note: For variables indicating left-sided lesions, all individuals with the corresponding left-sided lesions were counted. The same counting method was applied to variables indicating right-side lesions. BME, bone marrow edema.
